# Full “Laplacianised” posterior naive Bayesian algorithm

**DOI:** 10.1186/1758-2946-5-37

**Published:** 2013-08-23

**Authors:** Hamse Y Mussa, John BO Mitchell, Robert C Glen

**Affiliations:** 1 Unilever Centre for Molecular Science Informatics, Department of Chemistry, Lensfield Road, Cambridge, CB2 1EW, UK; 2 EaStCHEM School of Chemistry and Biomedical Sciences Research Complex, University of St Andrews, North Haugh, St Andrews, Scotland, KY16 9ST, UK

**Keywords:** Naive Bayes, Laplacian Corrected Modified Naive Bayes, Classifications, Cheminformatics

## Abstract

**Background:**

In the last decade the standard Naive Bayes (SNB) algorithm has been widely employed in multi–class classification problems in cheminformatics. This popularity is mainly due to the fact that the algorithm is simple to implement and in many cases yields respectable classification results. Using clever heuristic arguments “anchored” by insightful cheminformatics knowledge, Xia *et al.* have simplified the SNB algorithm further and termed it the Laplacian Corrected Modified Naive Bayes (LCMNB) approach, which has been widely used in cheminformatics since its publication.

In this note we mathematically illustrate the conditions under which Xia *et al.*’s simplification holds. It is our hope that this clarification could help Naive Bayes practitioners in deciding when it is appropriate to employ the LCMNB algorithm to classify large chemical datasets.

**Results:**

A general formulation that subsumes the simplified Naive Bayes version is presented. Unlike the widely used NB method, the Standard Naive Bayes description presented in this work is discriminative (not generative) in nature, which may lead to possible further applications of the SNB method.

**Conclusions:**

Starting from a standard Naive Bayes (SNB) algorithm, we have derived mathematically the relationship between Xia *et al.*’s ingenious, but heuristic algorithm, and the SNB approach. We have also demonstrated the conditions under which Xia *et al.*’s crucial assumptions hold. We therefore hope that the new insight and recommendations provided can be found useful by the cheminformatics community.

## Background

Broadly speaking there are two conceptually different ways to solve statistical problems: the frequentist and the Bayesian approaches. On the pros and cons of each method there are numerous excellent review articles and text books, such as the recent book by Murphy [1]. Unlike the frequentist approach, in the Bayesian approach any a priori knowledge about the probability distribution function that one assumes might have generated the given data (in the first place) can be taken into account when estimating this distribution function from the data at hand. If the data are noise–free and “complete”, the role of the a priori information in estimating the distribution function diminishes drastically. However, the a priori information can be crucial when the data are noisy and sparse. The latter scenario is typical in realistic large chemical datasets, which, arguably, makes Bayesian based statistics a powerful data analysis tool.

Unfortunately, Bayesian statistics in its fullest form is not computationally feasible in realistic cheminformatics data analyses. However, in recent years, a simplified version of the Bayesian approach, which is commonly known as the “Naive” Bayesian algorithm, has been found to be a useful classification tool in multi–class classification problems in cheminformactics. To this end a Naive Bayesian classifier is built on binary descriptor space. The descriptors/features *x*_*j*_, representing the compounds to be classified, assume binary values 0 or 1, where (*j* = 1,2,...,*L*) and *L* can typically be more than 1,000. Thus for some cheminformatics practitioners even the Naive Bayesian algorithm in its standard form is computationally prohibitive when the dataset is large. In this regard, Xia *et al*. [2] proposed a simpler version of the standard Naive Bayesian algorithm, albeit for binary classification problems; slight variants of this algorithm for multi–class classification can also be found in [3,4]. According to Rogers *et al.*[5], Rogers being a co–author of the work presented in [2], “the standard Naive Bayes was modified by considering only the effect of the presence of a feature and not its absence”. There are also a few more noticeable aspects of this proposed simplification: (a) the authors cleverly estimate directly – albeit heuristically – the a posteriori class probability for the present feature; (b) these authors (rather ingeniously) incorporate a Laplacian–correction into the estimated posterior class probability; and (c) the authors deem absent features not discriminating enough and therefore discard their contributions to the estimation of the posterior class. More than anything else it is this omission of the absent features from the Standard Naive Bayes (SNB) algorithm that makes Xia *et al.*’s proposed Naive Bayes Algorithm, termed Laplacian Corrected Modified Naive Bayes (LCMNB), (and its variants by different groups) computationally fast.

It is these three points, (a), (b) and (c), that we expound on in a mathematical setting to demonstrate under which conditions they hold – not only in an abstract sense, but also in the practical sense for a NB practitioner to make an informed decision as to when it is appropriate to employ SNB or LCMNB, in the cheminformatics context.

## Methods

### Naive Bayes

From Bayes’ theorem recall that [6]:

(1) $$\frac{p(\omega_{i}|\text{x})}{p(\omega_{i})}=\frac{p(\text{x}|\omega_{i})}{p(\text{x})}$$

where **x** = (*x*_1_,*x*_2_,...,*x*_*L*_) and *ω*_*i*_ denote the feature vectors and class labels, respectively; *x*_*j*_ and *L* being as described before, whereas *i* is just an index for the class labels. The terms *p*(*ω*_*i*_|**x**), *p*(**x**|*ω*_*i*_), *p*(*ω*_*i*_), and *p*(**x**) refer to the posterior probability for *ω*_*i*_ given **x**, the descriptor vector distribution conditioned on class *ω*_*i*_, the a priori probability of class *ω*_*i*_ occurring, and the descriptor vector density function, respectively – for more details, see ref. [3,4,6].

The left hand side of Eq. 1 can be expressed as follows [1,7]

(2) $$\frac{p(\omega_{i}|\text{x})}{p(\omega_{i})}=\frac{p(\omega_{i}|x_{1},x_{2},\mathrm{...},x_{L})}{p(\omega_{i})}$$

By virtue of Bayes’ theorem *p*(*ω*_*i*_|*x*_1_,*x*_2_,...,*x*_*L*_) can be rewritten as

(3) $$p(\omega_{i}|x_{1},x_{2},\mathrm{...},x_{L})=\frac{p(x_{1},x_{2},\mathrm{...},x_{L}|\omega_{i})p(\omega_{i})}{p(x_{1},x_{2},\mathrm{...},x_{L})}$$

which in turn allows us to rewrite Eq. 2 as

(4) $$\frac{p(\omega_{i}|\text{x})}{p(\omega_{i})}=\frac{p(\omega_{i})p(x_{1},x_{2},\mathrm{...},x_{L}|\omega_{i})}{p(\omega_{i})p(x_{1},x_{2},\mathrm{...},x_{L})}=\frac{p(x_{1},x_{2},\mathrm{...},x_{L}|\omega_{i})}{p(x_{1},x_{2},\mathrm{...},x_{L})}$$

Making use of the chain rule of probability [1,8], we can express *p*(*x*_1_,*x*_2_,...,*x*_*L*_|*ω*_*i*_) as

(5) $$\begin{array}{l} p(x_{1},x_{2},\mathrm{...},x_{L}|\omega_{i})=p(x_{1}|\omega_{i})p(x_{2}|\omega_{i},x_{1})\mathrm{.....}\\ \;p(x_{L}|\omega_{i},x_{1},x_{2},\mathrm{...},x_{L-1}) \end{array}$$

Plugging the right hand side of the equation above into Eq. 4 results in

(6) $$\frac{p(\omega_{i}|\text{x})}{p(\omega_{i})}=\frac{p(x_{1}|\omega_{i})p(x_{2}|\omega_{i},x_{1})\mathrm{.....p}(x_{L}|\omega_{i},x_{1},x_{2},\mathrm{...},x_{L-1})}{p(x_{1},x_{2},\mathrm{...},x_{L})}$$

In practice, it is extremely difficult to estimate *p*(*ω*_*i*_|**x**) or *p*(**x**|*ω*_*i*_). This reality inevitably forces one to make concessions over the degree of accuracy the estimated *p*(*ω*_*i*_|**x**) or *p*(**x**|*ω*_*i*_) can deliver. One widely employed scheme to obtain these probability distributions with compromised accuracy is to assume that individual descriptors *x*_*j*_, *j* = 1,2,...,*L*, are independent conditional on *ω*_*i*_. It is this naive assumption of independence among features to which the term “Naive” in “Naive Bayesian” refers.

Under this naive assumption, in Eq. 6, *p*(*x*_2_|*ω*_*i*_) = *p*(*x*_2_|*ω*_*i*_,*x*_1_), *p*(*x*_3_|*ω*_*i*_) = *p*(*x*_3_|*ω*_*i*_,*x*_1_,*x*_2_),..., *p*(*x*_*L*_|*ω*_*i*_) = *p*(*x*_*L*_|*ω*_*i*_,*x*_1_,*x*_2_...,*x*_*L*−1_). Thus, Eq. 6 modifies to

(7) $$\frac{p(\omega_{i}|\text{x})}{p(\omega_{i})}=\frac{p(x_{1}|\omega_{i})p(x_{2}|\omega_{i})\mathrm{...}p(x_{L}|\omega_{i})}{p(x_{1},x_{2},\mathrm{...},x_{L})}$$

Multiplying top and bottom of Eq. 7 by $p^{L}(\omega_{i})\Pi_{j=1}^{L}p(x_{j})$ yields

(8) $$\begin{array}{ll} \;\frac{p(\omega_{i}|\text{x})}{p(\omega_{i})} & =\frac{p^{L}(\omega_{i})p(x_{1}|\omega_{i})p(x_{2}|\omega_{i})\mathrm{...}p(x_{L}|\omega_{i})\Pi_{j=1}^{L}p(x_{j})}{p^{L}(\omega_{i})p(x_{1},x_{2},\mathrm{...},x_{L})\Pi_{j=1}^{L}p(x_{j})}\; \end{array}$$

(9) $$\begin{array}{ll} & \;=\frac{p^{L}(\omega_{i})p(x_{1}|\omega_{i})p(x_{2}|\omega_{i})\mathrm{...}p(x_{L}|\omega_{i})\Pi_{j=1}^{L}p(x_{j})}{\Pi_{j=1}^{L}p(x_{j})p^{L}(\omega_{i})p(x_{1},x_{2},\mathrm{...},x_{L})}\; \end{array}$$

Then making use of the fact that $p(\omega_{i}|x_{1})=\frac{p(\omega_{i})p(x_{1}|\omega_{i})}{p(x_{1})}$, $p(\omega_{i}|x_{2})=\frac{p(\omega_{i})p(x_{2}|\omega_{i})}{p(x_{2})}$,..., $p(\omega_{i}|x_{L})=\frac{p(\omega_{i})p(x_{L}|\omega_{i})}{p(x_{1})}$, we can rewrite Eq. 9 as

(10) $$\begin{array}{l} \frac{p(\omega_{i}|\text{x})}{p(\omega_{i})}=\frac{p(\omega_{i}|x_{1})p(\omega_{i}|x_{2})\mathrm{...}p(\omega_{i}|x_{L})\Pi_{j=1}^{L}p(x_{j})}{p^{L}(\omega_{i})p(x_{1},x_{2},\mathrm{...},x_{L})}\; \end{array}$$

or more compactly as

(11) $$\frac{p(\omega_{i}|\text{x})}{p(\omega_{i})}=\frac{\Pi_{j=1}^{L}p(\omega_{i}|x_{j})}{p^{L}(\omega_{i})}\times \frac{\Pi_{j=1}^{L}p(x_{j})}{p(x_{1},x_{2},\mathrm{....},x_{L})}$$

Clearly $\frac{\Pi_{j=1}^{L}p(x_{j})}{p(x_{1},x_{2},\mathrm{....},x_{L})}$ is common to all classes and therefore plays no role in classification. Thus, in practice (in the Naive Bayes context with which this work is concerned) one is required to estimate *p*(*ω*_*i*_|*x*_*j*_) and *p*(*ω*_*i*_).

Since generative approaches can be informative and “simpler” than their discriminative counterparts [9], we make use of Bayes’ theorem again, i.e., $p(\omega_{i}|x_{j})=\frac{p(\omega_{i})p(x_{j}|\omega_{i})}{p(x_{j})}$ and then estimate *p*(*ω*_*i*_|*x*_*j*_) through $\frac{p(\omega_{i})p(x_{j}|\omega_{i})}{p(x_{j})}$, where $p(x_{j})=\overset{C}{\underset{i=1}{\sum }}p(x_{j}|\omega_{i})p(\omega_{i})$ with *C* referring to the number of classes. *p*(*ω*_*i*_) denotes the a priori class probability, which is relatively easy to estimate. Thus, in our Bayesian context, the estimation of *p*(*ω*_*j*_|*x*_*j*_) boils down in practice to estimating *p*(*x*_*j*_|*ω*_*i*_).

### Estimation of *p*(*x*_*j*_|*ω*_*i*_), with *x*_*j*_ = 1 *and* 0

*p*(*x*_*j*_|*ω*_*i*_) can be estimated using the given data and assuming a *Beta* distribution as an a priori distribution for *p*(*x*_*j*_|*ω*_*i*_) [10]. (There are other possible prior distributions from which one can choose, but we select the *Beta* distribution for reasons that will transpire later). As described in Appedix **A**, a *Beta* a priori distribution *Beta*(*α*_*i*_,*β*_*i*_) for *p*(*x*_*j*_|*ω*_*i*_) results in a *p*(*x*_*j*_|*ω*_*i*_) estimator in the form [11]:

(12) $$p(x_{j}=1|\omega_{i})=\frac{N_{\text{ij}}+\alpha_{i}}{N_{\omega_{i}}+\beta_{i}+\alpha_{i}}$$

and of course

(13) $$p(x_{j}=0|\omega_{i})=1-\frac{N_{\text{ij}}+\alpha_{i}}{N_{\omega_{i}}+\beta_{i}+\alpha_{i}}$$

where $N_{\omega_{i}}$ and *N*_*ij*_, respectively, denote the number compounds in class *ω*_*i*_, and number of compounds in this class with descriptor *x*_*j*_ assuming value 1. *β*_*i*_ and *α*_*i*_ are *Beta* distribution hyper–parameters per class and the valid range of values that these hyper–parameter can assume are as defined in Appendix **A**. When *α*_*i*_ and *β*_*i*_ equal 1, *α*_*i*_ and *β*_*i*_ + *α*_*i*_ in Eqs. 12–13 can be viewed as a “Laplacian correction”.

## Results and discussion

### Estimation of *p*(*ω*_*i*_|*x*_*j*_ = 1) and *p*(*ω*_*i*_|*x*_*j*_ = 0)

#### Estimation of *p*(*ω*_*i*_|*x*_*j*_ = 1): In Our Approach

##### Remark 1

Assume that we have *N* chemical compounds (and their activity labels) available for training, where $N_{\omega_{i}}$ of these compounds belong to class *ω*_*i*_.

##### Remark 2

Assume that the class a priori distribution is taken as $p(\omega_{i})=\frac{N_{\omega_{i}}}{N}$, where $N_{\omega_{i}}>>\alpha_{i}+\beta_{i}$ (which is a valid assumption as found in any realistic large chemical dataset).

By virtue of Remark 1 and Eq. 12, the estimate of *p*(*ω*_*i*_|*x*_*j*_ = 1) becomes

(14) $$p(\omega_{i}|x_{j}=1)=\frac{p(x_{j}=1|\omega_{i})p(\omega_{i})}{p(x_{j}=1)}=\frac{\frac{N_{\text{ij}}+\alpha_{i}}{N_{\omega_{i}}+\alpha_{i}+\beta_{i}}\times \frac{N_{\omega_{i}}}{N}}{\overset{C}{\underset{i=1}{\sum }}\frac{N_{\text{ij}}+\alpha_{i}}{N_{\omega_{i}}+\alpha_{i}+\beta_{i}}\times \frac{N_{\omega_{i}}}{N}}$$

(recall that $p(x_{j})=\overset{C}{\underset{i=1}{\sum }}p(x_{j}|\omega_{i})p(\omega_{i})$).

Because of Remark 2, Eq. 14 can be simplified to

(15) $$p(\omega_{i}|x_{j}=1)=\frac{N_{\text{ij}}+\alpha_{i}}{\overset{C}{\underset{i=1}{\sum }}N_{\text{ij}}+\overset{C}{\underset{i=1}{\sum }}\alpha_{i}}=\frac{N_{\text{ij}}+\alpha_{i}}{N_{j}^{+}+\overset{C}{\underset{i=1}{\sum }}\alpha_{i}}$$

where $N_{j}^{+}$ is the number of times *x*_*j*_ assumes the value 1.

#### Estimation of *p*(*ω*_*i*_|*x*_*j*_ = 1): In Xia *et al.*’s Formulation

In the approach of Xia *et al.*, *p*(*ω*_*i*_|*x*_*j*_ = 1) is estimated as

(16) $$p(\omega_{i}|x_{j}=1)=\frac{N_{\text{ij}}+A_{i}}{N_{j}^{+}+K}$$

where *K* is as defined in Xia *et al.* and in their paper *A*_*i*_ is given as

() $$A_{i}=p(\omega_{i})\times K,\;\text{with}\;K=\overset{C}{\underset{i=1}{\sum }}A_{i}\;\text{as}\;\overset{C}{\underset{i=1}{\sum }}p(\omega_{i})=1$$

Eq. 16 constitutes what Xia *et al.* term “the Laplacian–Corrected Modified Naive Bayes (LCMNB)” estimator for *p*(*ω*_*i*_|*x*_*j*_ = 1).

If *α*_*i*_ in Eq. 15 is set to *A*_*i*_, Eq. 15 is exactly equivalent to Xia *et al.*’s estimator for *p*(*ω*_*i*_|*x*_*j*_ = 1) as can be seen in Eq. 16.

We note in passing that in Xia *et al.*’s case, *C* = 2 and $p(\omega_{2})=\frac{1}{K}$, which in their nomenclature denoted by *p*(*Active*) – that is, *A*_2_ = 1 while *A*_1_ = *K* − 1.

Initially we employed the *Beta* a priori distribution for the class conditional distribution to ascertain the equivalence of Eqs. 15 and 16. Fortunately, however, we have ended up with the general equations (Eqs. 14 – 15) that not only encapsulate the LCMNB scheme of Xia *et al.*, but also subsume the other various variants of LCMNB, such as those discussed in Nidhi *et al.* and Nigsch *et al.*’s papers [3,4].

At any rate, let us proceed to the nub of this work: Identifying the conditions under which the LCMNB algorithm holds with respect to the SNB algorithm. But first we need to describe the estimation of *p*(*ω*_*i*_|*x*_*j*_ = 0).

#### Estimation of *p*(*ω*_*i*_|*x*_*j*_ = 0): In Our Approach

In regard to the case of *x*_*j*_ = 0, we make use of Remark 1, Remark 2 and Eq. 13, which yield an estimator for *p*(*ω*_*i*_|*x*_*j*_ = 0) as

(17) $$p(\omega_{i}|x_{j}=0)=\frac{N_{\omega_{i}}-(N_{\text{ij}}+\alpha_{i})}{N-(N_{j}^{+}+\overset{C}{\underset{i=1}{\sum }}\alpha_{i})}$$

### Naive Bayes: scoring function from

For notational convenience let us denote $\frac{N_{\text{ij}}+\alpha_{i}}{N_{j}^{+}+\overset{C}{\underset{i=1}{\sum }}\alpha_{i}}$ in Eq. 15 and $\frac{N_{\omega_{i}}-(N_{\text{ij}}+\alpha_{i})}{N-(N_{j}^{+}+\overset{C}{\underset{i=1}{\sum }}\alpha_{i})}$ in Eq. 17 by *ξ*_*ij*_ and *ν*_*ij*_, respectively.

Thus, *p*(*ω*_*i*_|*x*_*j*_ = 1) and *p*(*ω*_*i*_|*x*_*j*_ = 0) may be written more succinctly as $p(\omega_{i}|x_{j})=\xi_{\text{ij}}^{x_{j}}\nu_{\text{ij}}^{\left(1-x_{j}\right)}$, which allows us to express Eq. 11 more compactly as

(18) $$\begin{array}{lll} \;\frac{p(\omega_{i}|\text{x})}{p(\omega_{i})} & =\frac{\Pi_{j}p(\omega_{i}|x_{j})}{p^{L}(\omega_{i})}\times \frac{\Pi_{j}p(x_{j})}{p(x_{1},x_{2},\mathrm{....},x_{L})}\;\\ & =\frac{\Pi_{j}\xi_{\text{ij}}^{x_{j}}\nu_{\text{ij}}^{\left(1-x_{j}\right)}}{p^{L}(\omega_{i})}\times \frac{\Pi_{j}p(x_{j})}{p(x_{1},x_{2},\mathrm{....},x_{L})}\; & \; \end{array}$$

Now we come to the core of this work, under which conditions does the LCMNB algorithm hold with respect to the SNB algorithm? Before we answer this question, we deem it instructive and more insightful to map Eq. 18 monotonically to a discriminant function, a “scoring function” (so to speak).

To this end, taking the logarithm of Eq. 18 results in

(19) $$\begin{array}{l} S_{\omega_{i}}(\text{x})=\text{ln}\frac{p(\omega_{i}|\text{x})}{p(\omega_{i})}=\text{ln}\frac{\Pi_{j}\xi_{\text{ij}}^{x_{j}}\nu_{\text{ij}}^{\left(1-x_{j}\right)}}{p^{L}(\omega_{i})}\times \frac{\Pi_{j}p(x_{j})}{p(x_{1},x_{2},\mathrm{...},x_{L})}\; \end{array}$$

(20) $$\begin{array}{lll} & \;=\underset{j}{\sum }x_{j}\;\text{ln}\;\xi_{\text{ij}}+\underset{j}{\sum }(1-x_{j})\;\text{ln}\;\nu_{\text{ij}}-L\times \;\text{ln}\;p(\omega_{i})\;\\ & \;\;\;\;\;\;\;+\underset{j}{\sum }\;\text{ln}\;p(x_{j})-\;\text{ln}\;p(x_{1},x_{2},\mathrm{...},x_{L})\; & \; \end{array}$$

Self–evidently, the term $\left[\underset{j}{\sum }\;\text{ln}\;p(x_{j})-\;\text{ln}\;p(x_{1},x_{2},\mathrm{...},x_{L})\right]$ is common to all classes and therefore does not play any role in classifying a given new compound. In other words, for practical classification purposes we are only interested in class dependent terms, i.e.,

(21) $$D_{\omega_{i}}(\text{x})=\underset{j}{\sum }\;x_{j}\;\text{ln}\;\xi_{\text{ij}}-\;\text{ln}\;p(\omega_{i})+\;\underset{j}{\sum }(1-x_{j})\;\text{ln}\;\nu_{\text{ij}}-(L-1)\times \;\text{ln}\;p(\omega_{i})$$

where $S_{\omega_{i}}(\text{x})=D_{\omega_{i}}(\text{x})+\left[\underset{j}{\sum }\;\text{ln}\;p(x_{j})-\;\text{ln}\;p(x_{1},x_{2},\mathrm{...},x_{L})\right]$

### Conditions

In Xia *et al.*’s approach, the LCMNB algorithm, is none other than $\sum_{j}x_{j}\;\text{ln}\;\xi_{\text{ij}}-\;\text{ln}\;p(\omega_{i})$ in Eq. 21. This means that in Xia *et al.*’s scheme the contributions from the terms depending on *x*_*j*_ = 0 for a given class, i.e.,

(22) $$\underset{j}{\sum }(1-x_{j})\;\text{ln}\;\nu_{\text{ij}}-(L-1)\times \;\text{ln}\;p(\omega_{i}),\;\forall i,\;i=1,2,\mathrm{..},C$$

are discarded. To the best of our knowledge, neither in Xia *et al.* nor in any other paper on the LCMNB approach has it been demonstrated that (i) the contribution of Eq. 22 is zero, i.e.,

(23) $$\underset{j}{\sum }(1-x_{j})\;\text{ln}\;\nu_{\text{ij}}-(L-1)\times \;\text{ln}\;p(\omega_{i})=0,\;\forall i,\;i=1,2,\mathrm{..},C$$

equally, in these papers, it has not been shown that (ii)

(24) $$\begin{array}{ll} |\underset{j}{\sum }x_{j}\;\text{ln}\;\xi_{\text{ij}} & -\;\text{ln}\;p(\omega_{i})|\;>>\;|\underset{j}{\sum }(1-x_{j})\;\text{ln}\;\nu_{\text{ij}}-(L-1)\\ & \times \;\text{ln}\;p(\omega_{i})|,\;\forall i,\;i=1,2,\mathrm{..},C \end{array}$$

nor has it been established that (iii)

(25) $$\underset{j}{\sum }(1-x_{j})\;\text{ln}\;\nu_{\text{ij}}-(L-1)\times \;\text{ln}\;p(\omega_{i})=\;\text{constant},\forall i,\;i=1,2,\mathrm{..},C$$

Thus unless one (or more) of the above – (i), (ii) and (iii) – is (are) met, the assumption on which the Modified Naive Bayesian algorithm is based is questionable and therefore its practitioners should pay attention to this discrepancy; clearly it is not justifiable to discard from the onset the contribution of $\underset{j}{\sum }(1-x_{j})\;\text{ln}\;\nu_{\text{ij}}-(L-1)\times \;\text{ln}\;p(\omega_{i})$ simply because features x _*j*_ are absent, i.e. *x*_*j*_ = 0.

For completeness, we consider also the case of the highly popular class prior distribution, $p(\omega_{i})=\frac{1}{C}$, i.e. *p*(*ω*_1_) = *p*(*ω*_2_) = ... = *p*(*ω*_*C*_). We hasten to add that this option was not included in the LCMNB scheme. At any rate, by simply repeating the arguments in the preceding sections, it is straightforward to show that one ends up with Eq. 21. In this scenario, though, *L* × ln*p*(*ω*_*i*_) is common to all classes and therefore does not play a role in classifying a new compound, i.e., $D_{\omega_{i}}(\text{x})$ reduces to

(26) $$D_{\omega_{i}}(\text{x})=\underset{j}{\sum }x_{j}\;\text{ln}\;\xi_{\text{ij}}+\underset{j}{\sum }(1-x_{j})\;\text{ln}\;\nu_{\text{ij}}$$

## Conclusions

Starting from a standard Naive Bayes (SNB) algorithm, we have derived mathematically the relationship between Xia *et al.*’s ingenious, but heuristic algorithm, and the standard Naive Bayes approach. We also describe the conditions on which Xia *et al.*’s crucial assumption – contributions from absent feature can be discarded – holds. It is our hope that, with this new insight, cheminformaticians may now be able to efficiently use the Modified version of the standard Naive Bayes algorithm, as proposed by Xia *et al.*, and subsequently by Nidhi *et al.* and Nigsch *et al*.

## Appendix

### Appendix A: Estimator of *p*(*x*_*j*_|*ω*_*i*_)

Here we give for completeness the proof that a priori *Beta* distribution leads to Eqs. 12 and 13 in the text.

For bookkeeping: 

*ω*_*i*_: class label indexed by *i*, *i* = 1,2,...,*C*.

*C*: Number of classes.

$N_{\omega_{i}}$: Number of samples in class *ω*_*i*_.

*N*_*ij*_: Number of samples in class *ω*_*i*_ with feature *x*_*j*_ = 1, *j* = 1,2,...,*L*.

*L*: Number of features.

We state from the onset, in the following derivation we follow closely the descriptions given in ref. [10]. We also note, for clarity’s sake, in the following analyses we abuse notation and use *x*_*jk*_ for both the random variable and its realization.

In this work, **x** ∈ {0,1}^*L*^, i.e. *x*_*j*_ ∈ {0,1} and suppose that *x*_*j*_ are independent Bernoulli random variables (and this is in fact the assumption made in the Naive Bayesian approach). Thus, in the Naive Bayesian setting *p*(**x**|*ω*_*i*_) can be given as

(27) $$p(\text{x}|\omega_{i})=\Pi_{j=1}^{L}\text{Ber}(x_{j}|\mu_{\text{ij}})=\Pi_{j=1}^{L}\mu_{\text{ij}}^{x_{j}}{\left(1-\mu_{\text{ij}}\right)}^{1-x_{j}}$$

where *μ*_*ij*_ is an estimate for the conditional probability that feature *j* occurs in class *ω*_*i*_, and is what we are trying to estimate given a set of compounds assumed to belong to class *ω*_*i*_. (In our context, *μ*_*ij*_ is an estimator for *p*(*x*_*j*_|*ω*_*i*_), where *p*(*x*_*j*_|*ω*_*i*_)is as defined in the text.)

To estimate *μ*_*ij*_ in a Bayesian framework, we first view *μ*_*ij*_ as a random variable, then choose an “appropriate” prior and likelihood for the random variable *μ*_*ij*_.

Let us suppose that our a priori knowledge about the random variable *μ*_*ij*_ indicates that *μ*_*ij*_ is described by a *Beta* distribution, i.e.,

(28) $$\begin{array}{ll} \;\pi (\mu_{\text{ij}})= & \frac{1}{B(\alpha_{i},\beta_{i})}\mu_{\text{ij}}^{\alpha_{i}-1}{\left(1-\mu_{\text{ij}}\right)}^{\beta_{i}-1},0\;\leq \mu_{\text{ij}}\leq 1,\alpha_{i},\\ & \;\beta_{i}>0,\;i=1,2,\mathrm{...}C \end{array}$$

where *B*(*α*_*i*_,*β*_*i*_) ensures that the *Beta* distribution is normalised

Using the Bayes’ theorem, then the posterior probability for *μ*_*ij*_ on the training data can be given by

(29) $$\begin{array}{l} \pi (\mu_{\text{ij}}|x_{j1},x_{j2},\mathrm{...},x_{{\text{jN}}_{\omega_{i}}})=\frac{f(x_{j1},x_{j2},\mathrm{...},x_{{\text{jN}}_{\omega_{i}}}|\mu_{\text{ij}})\pi (\mu_{\text{ij}})}{\int_{0}^{1}f(x_{j1},x_{j2},\mathrm{...},x_{{\text{jN}}_{\omega_{i}}}|\mu_{\text{ij}})\pi (\mu_{\text{ij}})d\mu_{\text{ij}}} \end{array}$$

where $f(x_{j1},x_{j2},\mathrm{...},x_{{\text{jN}}_{\omega_{i}}}|\mu_{\text{ij}})$refers to the likelihood, and *x*_*j*1_,*x*_*j*2_,... and $x_{{\text{jN}}_{\omega_{i}}}$ denote the *j*^*th*^ feature of the $N_{\omega_{i}}$ samples/compounds from class *ω*_*i*_. As the samples are assumed independent, then $f(x_{j1},x_{j2},\mathrm{...},x_{{\text{jN}}_{\omega_{i}}}|\mu_{\text{ij}})$ becomes $\Pi_{k=1}^{N_{\omega_{i}}}f(x_{\text{jk}}|\mu_{\text{ij}})=\Pi_{k=1}^{N_{\omega_{i}}}\mu_{\text{ij}}^{x_{\text{jk}}}{\left(1-\mu_{\text{ij}}\right)}^{1-x_{\text{jk}}}$, *i.e.*

(30) $$\Pi_{k=1}^{N_{\omega_{i}}}f(x_{\text{jk}}|\mu_{\text{ij}})=\mu_{\text{ij}}^{\sum_{k=1}^{m}x_{\text{jk}}}{\left(1-\mu_{\text{ij}}\right)}^{N_{\omega_{i}}-\sum_{k=1}^{N_{\omega_{i}}}x_{\text{jk}}}$$

Thus, the posterior *π*(*μ*_*ij*_|*x*_*j*1_,*x*_*j*2_,...,*x*_*jM*_) in Eq. 29 modifies to

(31) $$\begin{array}{l} \pi (\mu_{\text{ij}}|x_{j1},x_{j2},\mathrm{...},x_{{\text{jN}}_{\omega_{i}}})\\ \;=\frac{\mu_{\text{ij}}^{\sum_{k=1}^{N_{\omega_{i}}}x_{\text{jk}}}{\left(1-\mu_{\text{ij}}\right)}^{N_{\omega_{i}}-\sum_{k=1}^{N_{\omega_{i}}}x_{\text{jk}}}\pi (\mu_{\text{ij}})}{\int_{0}^{1}\mu_{\text{ij}}^{\sum_{k=1}^{N_{\omega_{i}}}x_{\text{jk}}}{\left(1-\mu_{\text{ij}}\right)}^{N_{\omega_{i}}-\sum_{k=1}^{N_{\omega_{i}}}x_{\text{jk}}}\pi (\mu_{\text{ij}})d\mu_{\text{ij}}} \end{array}$$

i.e.,

(32) $$\begin{array}{l} \pi (\mu_{\text{ij}}|x_{j1},x_{j2},\mathrm{...},x_{{\text{jN}}_{\omega_{i}}})\propto \mu_{\text{ij}}^{\sum_{k=1}^{N_{\omega_{i}}}x_{\text{jk}}}{\left(1-\mu_{\text{ij}}\right)}^{N_{\omega_{i}}-\sum_{k=1}^{N_{\omega_{i}}}x_{\text{jk}}}\pi (\mu_{\text{ij}})\; \end{array}$$

(33) $$\begin{array}{lll} & \;=\mu_{\text{ij}}^{\sum_{k=1}^{N_{\omega_{i}}}x_{\text{jk}}}{\left(1-\mu_{\text{ij}}\right)}^{N_{\omega_{i}}-\sum_{k=1}^{N_{\omega_{i}}}x_{\text{jk}}}\; & \;\\ & \;\;\times \mu_{\text{ij}}^{\alpha_{i}-1}{\left(1-\mu_{\text{ij}}\right)}^{\beta_{i}-1}\; \end{array}$$

(34) $$\begin{array}{ll} & \;=\mu_{\text{ij}}^{N_{\text{ij}}+\alpha_{i}-1}{\left(1-\mu_{\text{ij}}\right)}^{N_{\omega_{i}}-N_{\text{ij}}+\beta_{i}-1}\; \end{array}$$

Clearly, in Eq. 34, the posterior density for *μ*_*ij*_ given the samples $x_{j1},x_{j2},\mathrm{...},x_{{\text{jN}}_{\omega_{i}}}$ has the same form as the prior for *μ*_*ij*_[11], i.e.,

(35) $$\begin{array}{l} \pi (\mu_{\text{ij}}|x_{j1},x_{j2},\mathrm{...},x_{{\text{jN}}_{\omega_{i}}})\\ \;=\frac{1}{B(N_{\text{ij}}+\alpha_{i},N_{\omega_{i}}-N_{\text{ij}}+\beta_{i})}\mu_{\text{ij}}^{N_{\text{ij}}+\alpha_{i}-1}{\left(1\;-\;\mu_{\text{ij}}\right)}^{N_{\omega_{i}}-N_{\text{ij}}+\beta_{i}-1} \end{array}$$

which is none other than another *Beta* distribution. This means that the Bayes estimator of *μ*_*ij*_, which is the estimate we are interested in, is the mean of the posterior distribution obtained [11]:

(36) $$E[\mu_{\text{ij}}|x_{j1},x_{j2},\mathrm{...},x_{{\text{jN}}_{\omega_{i}}}]=\frac{N_{\text{ij}}+\alpha_{i}}{N_{\omega_{i}}+\alpha_{i}+\beta_{i}}$$

In other words,

(37) $$p(x_{j}|\omega_{i})=\frac{N_{\text{ij}}+\alpha_{i}}{N_{\omega_{i}}+\alpha_{i}+\beta_{i}}$$

*QED*.

An accessible description of the derivation of Eq. 37 can be found in ref. [10].

## Competing interests

The authors declare that they have no competing interests.

## Authors’ contributions

HYM conceived the idea that constitutes the nub of the presented work. This author also carried out the bulk of the mathematical derivations. RCG contributed to the Bayesian aspect of the work. JBOM conceptually contributed to the derivation given in Appendix A. The three authors participated in drafting the manuscript. All authors read and approved the final manuscript.
